# Supplementary figures and images for: Mechanical response characteristics of full depth reclaimed pavement based on 3D-move model
Source: PLoS One. 2025 May 8;20(5):e0323163. doi: 10.1371/journal.pone.0323163 (PMC12061192; doi:10.1371/journal.pone.0323163)

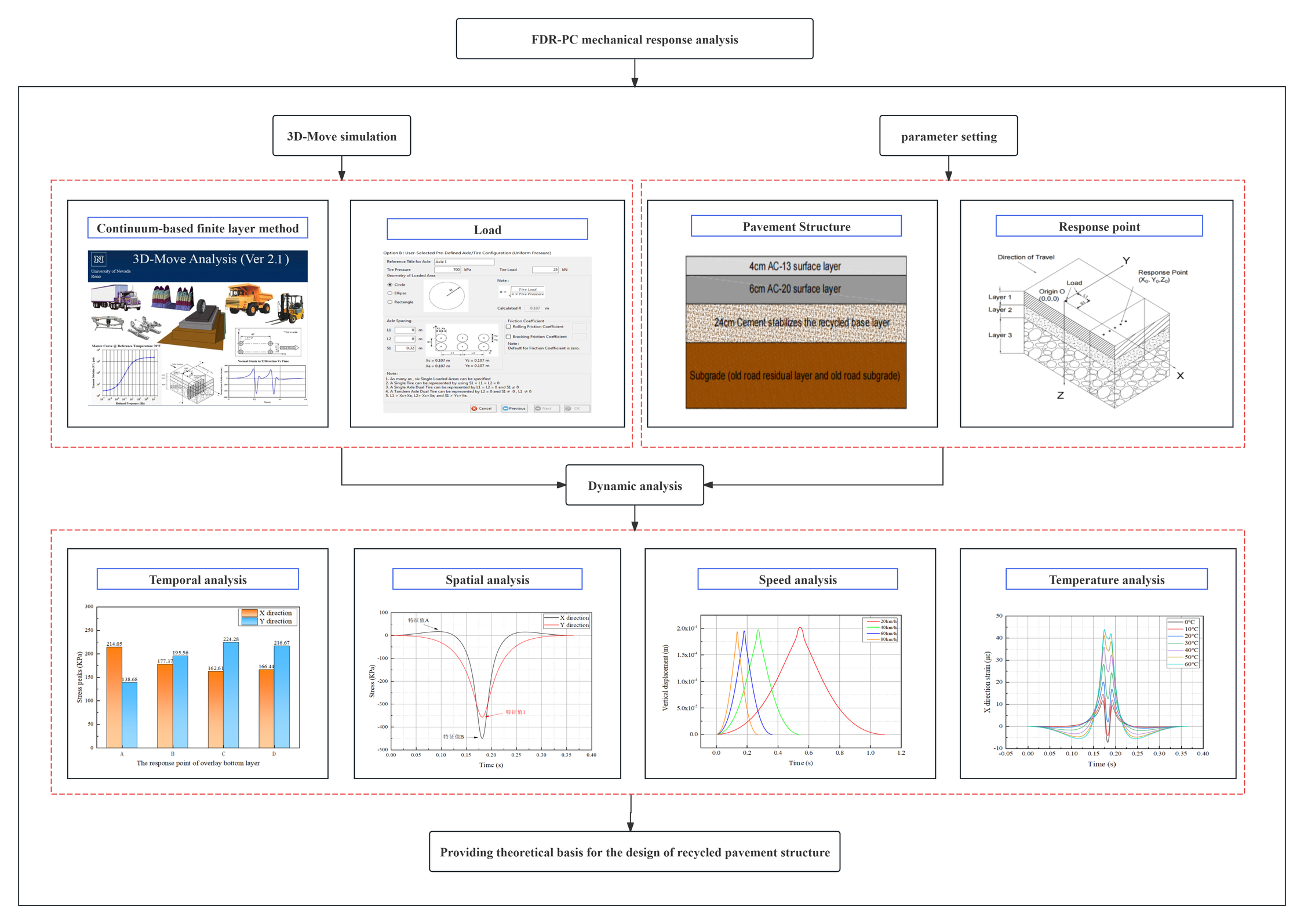

Supplement: S1 Fig — (TIF) [file pone.0323163.s001.tif]
