# Supplementary material for: Mechanical response characteristics of full depth reclaimed pavement based on 3D-move model
Source: PLoS One. 2025 May 8;20(5):e0323163. doi: 10.1371/journal.pone.0323163 (PMC12061192; doi:10.1371/journal.pone.0323163)
Supplement: Attachment — Submitted filename: Response to Reviewer 2 Comments.docx [file pone.0323163.s003.docx]

**Response to Reviewer 2 Comments**

Dear Reviewer #2,

We would like to express our sincere appreciation for your helpful comments to improve this paper. We have made corresponding revision according to your advice. Revised portion are marked in red in the paper. The following are answers and revisions in response to your suggestions.

**Comment 1:**

Incorrect use of punctuation between keywords.

**Response 1:**

Thank you for pointing out the issue regarding the incorrect use of punctuation between keywords. We have carefully reviewed and corrected these punctuation errors throughout the text to ensure proper grammar and readability.

**Comment 2:**

The order between ‘et al’ in the introduction and the number of the citation is not standardised.

**Response 2:**

Thank you for highlighting the issue regarding the non-standardised order between 'et al' and the citation number in the introduction. We have gone through the relevant parts and adjusted them to follow the correct standardised format.

**Comment 3:**

Are there any limitations of the finite layer method in the 3D-Move programme when dealing with complex pavement structures (e.g. where there is an inhomogeneous material distribution)?

**Response 3:**

Thank you for your incisive comment regarding the limitations of the finite layer method within the 3D-Move programme when handling complex pavement structures.

Indeed, the finite layer method in the 3D-Move programme has certain limitations when dealing with complex pavement structures characterized by inhomogeneous material distribution. However, in our current study, the pavement structure under consideration is relatively typical, mainly focusing on the mechanical responses of full-depth recycled pavements under common working conditions. In such scenarios where the pavement structure is layered and relatively homogeneous and our interest lies in the responses at specific positions, the finite layer method demonstrates a significant advantage in terms of computational efficiency.In view of the limitations of the model, the material parameters are simplified and assumed in order to meet the applicable conditions of the finite layer method.

**Comment 4:**

Chapter 4 section headings should be capitalised and the hierarchy confused.

**Response 4:**

Thank you for pointing out the issues regarding the capitalisation and hierarchy confusion of Chapter 4 section headings. We have carefully reviewed and made the necessary adjustments to capitalise the headings properly and clarify the hierarchical structure as per the required standards.

**Comment 5:**

In the dynamic analysis, is it sufficient to set the default travelling speed to 60km/h for the study? Should more speed conditions be added to the analysis because of the wide range of speed variations in different car models and actual road conditions?

**Response 5:**

Thank you for your astute comment regarding the setting of the travelling speed in the dynamic analysis.

In our study, the decision to set the default travelling speed at 60km/h was based on multiple rationales. Firstly, this speed aligns with the common design speeds of numerous typical roads. It represents a standard velocity that is frequently adopted in road engineering designs and is thus a reasonable benchmark for our analysis focused on understanding the general mechanical responses of the pavement under normal traffic conditions.

Secondly, it draws on the experience from a wealth of previous similar studies. Many prior investigations have utilized this or comparable speeds as a reference point to establish foundational knowledge about pavement mechanics under dynamic loads, and their findings have provided valuable references for our current research.

While it is undeniable that in real-world scenarios, different car models and actual road conditions lead to a wide range of speed variations, our extensive preliminary studies have shown that within a certain range of speeds, the influence trends of vehicle speed on the pavement's mechanical responses exhibit remarkable similarities. That is to say, although the specific magnitudes of the responses may vary with different speeds, the overall patterns and directions of change remain consistent. Therefore, choosing 60km/h as the default speed enables us to capture these general trends effectively and still gain valuable insights into the fundamental mechanical behavior of the pavement under dynamic loads.

**Comment 6:**

In the conclusion,“within a certain range of speed” does not specify the range. Add a specific range of speed.

**Response 6:**

Thank you for your comment regarding the specification of the speed range in the conclusion. We have taken your advice seriously and have now added “within the speed range of 20 km/h to 80 km/h” in the manuscript as you suggested.

**Comment 7:**

Numerical simulation has also been used to study the mechanical response of pavements, what are the specific aspects of this paper that are innovative in terms of research content and methodology?

**Response 7:**

Thank you for your insightful comment regarding the innovation aspects of our paper.

Our study indeed presents several innovative elements in both research content and methodology.

In terms of the research content, we specifically focus on the Full-Depth Recycled Pavement (FDR-PC). Currently, there is relatively limited research on the mechanical properties of this particular type of pavement. Our study fills this gap by providing crucial theoretical bases for its structural design and the selection of technical solutions. This enables a better understanding of how FDR-PC performs mechanically and offers valuable guidance for its practical application in the field of pavement engineering.

When it comes to the research methodology, we utilize the 3D-Move program to conduct a dynamic analysis of the pavement's mechanical response. What sets our approach apart is that we comprehensively consider four aspects, namely plane, space, speed, and temperature. Unlike previous studies that often focused on only a single factor or a limited number of factors, our multi-factor comprehensive analysis method is more holistic. By analyzing the characteristics of mechanical responses under different factors, we obtain richer and more comprehensive research results, which allows for a deeper exploration into the mechanical behavior of the Full-Depth Recycled Pavement.

We believe these innovative aspects contribute to the uniqueness and value of our study in the existing body of pavement research.
